# *Philodulcilactobacillus myokoensis* gen. nov., sp. nov., a fructophilic, acidophilic, and agar-phobic lactic acid bacterium isolated from fermented vegetable extracts

**DOI:** 10.1371/journal.pone.0286677

**Published:** 2023-06-21

**Authors:** Tomoaki Kouya, Yohei Ishiyama, Shota Ohashi, Ryota Kumakubo, Takeshi Yamazaki, Toshiki Otaki

**Affiliations:** 1 Department of Materials Chemistry and Bioengineering, National Institute of Technology, Oyama College, Oyama, Tochigi, Japan; 2 MIYATOU YASOU KENKYUJO CO., LTD., Niigata, Japan; Universidad Autonoma de Chihuahua, MEXICO

## Abstract

Lactic acid bacteria are commonly in the fermentation industry and pose potential positive effects on health. In this study, a new lactic acid bacterium was isolated from fermented vegetable extracts in Myoko, Niigata, Japan. This bacterium is fructophilic, acidophilic, and hard to grow on agar medium. The isolate is Gram-stain-positive, non-spore-forming, non-motile, rod-shaped, and catalase-negative. Growth occurred at pH 3.5–5.5, with optimal growth at pH 4.5–5.0. The cells formed colonies on a solid MRS medium with 20% (w/v) sucrose and 0.8% (w/v) gellan gum under anaerobic conditions. The bacterium was able to grow on up to 50% (w/v) sucrose but not on d-glucose. Moreover, 16S rRNA gene sequence analysis revealed that the strain was most closely related to *Apilactobacillus ozensis* (93.1% sequence similarity). The values of average nucleotide identity, digital DNA-DNA hybridization, average amino acid sequence identity, and amino acid identity of conserved genes were calculated between the isolated strain (type strain is WR16-4^T^ = NBRC 115064^T^ = DSM 112857^T^) and its phylogenetically closest type strains. The average nucleotide identity values (73.36–78.28%) and DNA-DNA hybridization values (16.3–32.9%) were significantly lower than the threshold values for species boundaries. The average amino acid sequence identity values (53.96–60.88%) were significantly below the threshold boundary of genus demarcation (68%). The amino acid identity of conserved genes values compared to strain WR16-4^T^ were the genera *Apilactobacillus*, *Nicoliella spurrieriana* SGEP1_A5^T^, *Acetilactobacillus jinshanensis* HSLZ-75^T^, and *Fructilactobacillus* were 62.51–63.79%, 62.87%, 62.03%, and 58.00–61.04%, respectively. The 16S rRNA gene and core genome phylogenetic trees suggested that this novel strain was most closely related to the type strain of *A*. *jinshanensis* HSLZ-75^T^. Based on the physiological, morphological, and phenotypical characteristics of strain WR16-4^T^, we propose its classification as a novel genus, *Philodulcilactobacillus myokoensis* gen. nov., sp. nov.

## Introduction

Lactic acid bacteria are found in numerous habitats, including the gastrointestinal tracts of animals, feces, plant flowers, fruits, sap, and various fermented foods [1–3]. Various lactic acid bacteria, such as *Lactobacillus* spp., *Lacticaseibacillus* spp., *Lactiplantibacillus* spp., *Leuconostoc* spp., and *Lactococcus* spp. are applied in the preparation of fermented vegetables, dairy products, and meat products. They are one of the most commonly used bacterial groups in the fermentation industry owing to their advantages in food preservation, improving taste and flavor, and as probiotics for human health [4]. However, lactic acid bacteria have complex nutritional requirements for growth, requiring the supply of carbohydrates, amino acids, peptides, fatty acid esters, salts, nucleic acid derivates, and vitamins [5, 6]. Furthermore, several lactic acid bacteria, such as halophilic lactic acid bacteria (e.g., *Tetragenococcus* sp.), lactic acid bacteria associated with malolactic fermentation (e.g., *Oenococcus* sp.), and sake and beer spoilage lactic acid bacteria, require specific environmental conditions for their isolation and cultivation [7–9].

Fructophilic lactic acid bacteria (FLAB), a recently discovered group, are found in flowers, fruits, fermented fruits, and insect gastrointestinal tracts [10]. FLAB are heterofermentative, producing several metabolites (lactic acid, acetic acid, mannitol, carbon dioxide, etc.) [11]. They have some unique biochemical characteristics, including a preference for d-fructose as a substrate and poor growth on d-glucose alone but enhanced growth in the presence of electron acceptors such as pyruvate, oxygen, and d-fructose [11, 12]. FLAB require these electron acceptors because they partially or completely lack *adhE*, which codes for the bifunctional alcohol/acetaldehyde dehydrogenase (AdhE). AdhE is an essential enzyme for maintaining the balance between NAD^+^ and NADH in the heterolactic phosphoketolase pathway [13–15].

In the present study, we report the discovery of a novel lactic acid bacterium isolated from fermented vegetable extracts (obtained from MIYATOU YASOU KENKYUJO CO., LTD., Myoko, Niigata, Japan). This strain grew slowly when the original extract was added to an MRS (deMan, Rogosa, and Sharpe) medium, and its existence was microscopically confirmed. However, cultivation of the bacterium under general culture conditions was challenging, and counting viable cells was not possible as colony formation was suppressed when the cells were grown on a solid agar plate. Therefore, the present study aimed to determine the morphological, physiological, and biochemical characteristics of this novel lactic acid bacterium using comprehensive analyses. Our findings provide a comprehensive basis for elucidating the characteristics of the novel isolated strain, especially its growth on sucrose.

## Materials and methods

### Isolation of strain WR16-4^T^ from fermented vegetable extracts

Strain WR16-4^T^ was isolated from a fermented vegetable extract (MIYATOU YASOU KENKYUJO CO., LTD., Myoko, Niigata, Japan). The extract was prepared as follows: cut papaya, apple, spinach, and white sugar were mixed at a ratio of 5:4:1:5 (w/w) and fermented at 20–25°C for 1 week.; the fermented product was pressed with a compressor and the extract was fermented under the same conditions for additional 7 weeks (a total of 8 weeks). Water was added to bring the Brix to 25%, and the yeast (*Clavispora* sp.) was inoculated and incubated at 20–25°C for 5 days while white sugar was gradually added (final Brix: approximately 50%). This solution was used as the starter culture. Cut Japanese white radish and white sugar were mixed at a ratio of 1:1 (w/w). Starter culture was added (about 2 mL/ kg-Japanese white radish) and fermented at 20–25°C for 1 week; the fermented product was pressed with a compressor and the extract was fermented under the same conditions for additional 7 weeks (a total of 8 weeks). Brix and the pH of the fermented Japanese white radish extract were approximately 50% and pH 3–4, respectively.

The following procedure was used for the enrichment culture and isolation of strain WR16-4^T^. The fermented extracts of each vegetable were prepared as described above and then mixed in the proportions shown in S1 Table. The stock solution of boiled wild grasses was prepared as follows: the dry materials shown in S2 Table were mixed with 600 L of water and boiled for 1 h, and the liquid was collected and further heated to double the concentration. The fermented vegetable extracts (10% (w/v)), the stock solution of boiled wild grasses (45% (w/v)), white sugar (45% (w/v)), and cycloheximide (final concentration: 10 ppm) were mixed and sterilized by autoclaving. This mixture was then used as an enrichment culture medium. The unsterilized fermented Japanese white radish extract was added to the culture medium and was enriched several times by the subsequent propagation of culture. MRS agar (pH 4.5) supplemented with 10% (v/v) fermented vegetable extracts and 10% (w/v) sucrose was sterilized by autoclaving and used as the isolation medium. The enriched culture mixed with the isolation medium was then poured on a Petri dish and incubated at 25°C for 10 d in an anaerobic environment. Colonies were selected from the agar plate and resuspended in a modified MRS medium (pH 4.5) containing (per L): 100 g sucrose (instead of 20 g d-glucose), 1.0 g Tween 80, 10 g fish meat extract, 10 g HIPOLYPEPTON (Nihon Pharmaceutical Co., Ltd., Tokyo, Japan), 5.0 g yeast extract, 5.0 g sodium acetate, 2.0 g K_2_HPO_4_, 2.0 g diammonium hydrogen citrate, 0.1 g MgSO_4_·7H_2_O, and 0.05 g MnSO_4_·5H_2_O. After cultivation at 30°C for 72 h, the cells were suspended in 10% (w/v) glycerol and stored at –80°C. All experiments were performed in accordance with the relevant guidelines and regulations.

### Colony-forming test on agar and gellan gum plates

Agar (15 g/L, FUJIFILM Wako Pure Chem. Corp., Osaka, Japan) or gellan gum (8 g/L, FUJIFILM Wako Pure Chem. Corp.) was used as a gelling agent in a solid MRS medium containing 20% (w/v) sucrose (pH 4.5). Cells from the late log to early stationary growth phase (grown anaerobically for 48 h) were diluted to an optical density at 660 nm (OD_660 nm_) of 1 (approximately 2.5 × 10^8^ cells/mL) with a distilled saline solution (0.85% (w/v) NaCl). Total cell counting was performed using a phase-contrast microscope (cells/mL). The number of colony-forming cells (CFU/mL) was determined by the plate culture method. Diluted cultures (0.05 mL) were spread on both plates and incubated at 30°C for 4, 7, and 14 d under anaerobic and aerobic conditions. Data are averages of triplicate (± SD) determinations.

### Carbohydrate fermentation profile

Carbohydrate fermentation test was performed using API 50 CH strips (bioMérieux, Marcy-l’Étoile, France). API 50 CHL medium (pH 6.7–7.1) with bromocresol purple and modified medium (pH 5.0) composed of the same components but with bromocresol green as the pH indicator, were used for evaluation, and the cultures were incubated at 30°C for 120 h. Carbohydrate consumption was determined according to the manufacturer’s manual. Purple to yellow was recorded as positive, very light yellow was recorded as weakly positive, and no color change was recorded as negative in the API 50 CHL medium containing bromocresol purple. A similar evaluation was performed using the medium with a bromocresol green indicator, except that a color change from green to yellow was recorded as positive. All experiments were performed in triplicate.

### Scanning electron microscopy (SEM) analysis

SEM was performed using a scanning electron microscope (JSM-7800F, JEOL Ltd., Tokyo, Japan) with an acceleration voltage of 10 kV to observe the cell morphology. The strain was grown on an MRS medium containing 20% (w/v) sucrose (pH 4.5) at 30°C for 48 h. Cells were harvested by centrifugation (15,000×*g*, 4°C, 10 min), washed twice in 0.1 M phosphate buffer (pH 7.0), and fixed for 4 h with 2.0% glutaraldehyde in 0.1 M phosphate buffer (pH 7.0). Dehydration was performed in 50% (v/v), 70% (v/v), 90% (v/v), and 100% ethanol in ultrapure water with 15 min incubation for each step. The sample was then dipped into t-butyl alcohol, frozen to -20°C, and freeze-dried in a vacuum evaporator. The specimen was subsequently subjected to platinum coating using an auto fine coater (JEC-3000FC, JEOL Ltd.).

### Physiological, morphological, and biochemical characteristics

Gram staining and catalase activity were examined after anaerobic cultivation for 96 h on MRS medium (pH 4.5) containing 20% (w/v) sucrose and 0.8% (w/v) gellan gum. Cell motility was measured in a soft solid MRS medium (containing 20% (w/v) sucrose and 0.08% (w/v) gellan gum, pH 4.5). Spore formation was determined by heat-treatment of cells suspended in MRS medium at 80°C for 10 min. Cell growth was evaluated using d-glucose as a carbon source in an MRS medium (pH 4.5) with some electron acceptors (oxygen, 0.5% (w/v) pyruvate, and 0.5% (w/v) d-fructose) under anaerobic and aerobic conditions for 48 h and 120 h. Growth at 15, 30, and 45°C was evaluated in MRS medium (pH 4.5) containing 20% (w/v) sucrose for 48 h and 120 h. Moreover, salt tolerance was evaluated at 2% (w/v) NaCl in an MRS medium (pH 4.5) containing 20% (w/v) sucrose. Organic acid production was evaluated as follows: strain WR16-4^T^ was cultivated using 2.0% (w/v) d-glucose as a carbon source in MRS medium (pH 4.5) with 0.5% (w/v) d-fructose as an electron acceptor under anaerobic conditions for 72 h. The supernatant was obtained by centrifugation (15,000×*g*, 10 min) of culture broth. Glucose consumption was measured using a glucose oxidase-peroxidase kit (Glucose C-II Test Kit Wako; Wako Pure Chemical Industries, Osaka, Japan), and the remaining total sugar (d-glucose and d-fructose) was determined using the phenol sulfuric acid method. Organic acid concentrations were measured using an HPLC system (Shimadzu, Kyoto, Japan) [16]. The G+C content was calculated from the draft genome. The cellular fatty acid composition was analyzed according to the procedures in the manual of Sherlock Microbial Identification system (version 6.0). Cell wall amino acids were determined according to the method described by Uchida and Suzuki [17, 18].

### Effect of pH on growth

Strain WR16-4^T^ was pre-cultured at 30°C for 48 h, and 1 mL culture aliquots were centrifuged at 15,000 × *g* at 4°C for 10 min. The supernatant was removed, and the cell pellet was resuspended in an MRS medium containing 20% (w/v) sucrose (pH 3.0, 3.5, 4.0, 4.5, 5.0, 5.5, 6.0, and 6.5) at an initial OD_660 nm_ = 0.05–0.1. Bacterial growth (OD_660 nm_) was measured after 48 h cultivation in a 96-well microplate (200 μL/well) under anaerobic conditions. All experiments were performed in triplicate, and the data are expressed as mean ± SD.

### Growth on sucrose and carbohydrate combinations

For the sucrose consumption test, pre-cultured cells were centrifuged and resuspended in MRS medium (pH 4.5) containing several sucrose concentrations (5, 10, 20, 30, 40, 50, and 60% (w/v)) in a 96-well microplate. Bacterial growth (OD_660 nm_) was evaluated after 48 h cultivation under anaerobic conditions.

A carbohydrate combination test was performed using d-glucose, d-fructose, and sucrose separately or in combination. The carbohydrates were added to the medium at 20% (w/v) final concentration (20% (w/v) d-glucose (G20]), 20% (w/v) d-fructose (F20]), 20% (w/v) sucrose (S20]), 15% (w/v) d-fructose and 5% (w/v) d-glucose (F[15]+G[5]), 10% (w/v) d-fructose and 10% (w/v) d-glucose (F[10]+G[10]), or 5% (w/v) d-fructose and 15% (w/v) d-glucose (F[5]+G[15])). Other experimental conditions and measurements were the same as described above. All experiments were performed in triplicate, and the data are expressed as mean ± SD.

### Phylogenetic analysis

For comparative analysis using the strain WR16-4^T^, the 16S rRNA gene sequences and RefSeq data of the genome sequences of closely related lactic acid bacteria (15 species, *Nicoliella spurrieriana* SGEP1_A5^T^, *Acetilactobacillus jinshanensis* HSLZ-75^T^, *Apilactobacillus apisilvae* SG5_A10^T^, *Apilactobacillus ozensis* DSM 23829^T^, *Apilactobacillus micheneri* Hlig3^T^, *Apilactobacillus bombintestini* BHWM-4^T^, *Apilactobacillus apinorum* Fhon13^T^, *Apilactobacillus kunkeei* DSM 12361^T^, *Apilactobacillus nanyangensis* HN36-1^T^, *Apilactobacillus quenuiae* HV-6^T^, *Apilactobacillus timberlakei* HV-12^T^, *Fructilactobacillus fructivorans* CCUG 32260^T^, *Fructilactobacillus florum* DSM 22689^T^, *Fructilactobacillus lindneri* DSM 20690^T^, and *Fructilactobacillus sanfranciscensis* DSM 20451^T^), and one outgroup (*Lactiplantibacillus plantarum* subsp. *plantarum* JCM 1149^T^), were downloaded from the National Center for Biotechnology Information (https://www.ncbi.nlm.nih.gov/home/download/) and used.

The isolated strain was sent to FASMAC sequencing service (Fasmac, Kanagawa, Japan) for 16S rRNA gene sequence analysis. Similar sequences were obtained from the Basic Local Alignment Search Tool (BLAST; https://blast.ncbi.nlm.nih.gov/Blast.cgi). Multiple sequence alignments were performed using the Clustal W program [19]. Moreover, the MEGA X software (version 10.2.6) was used for the construction of phylogenetic trees from the aligned sequences [20]. The phylogenetic trees of 16S rRNA gene was built using the maximum-likelihood methods based on the Kimura two-parameter model with 1000 bootstrap replicates, and the genetic distances between the sequences were calculated.

The draft genome of strain WR16-4^T^ was sequenced using the PacBio^®^ Sequel IIe system (Pacific Bioscience, Menlo Park, CA, USA). A phylogenetic tree of the core genome was reconstructed based on concatenated multiple sequence alignments of 81 core bacterial genes using UBCG2 [21] to infer phylogenetic relationships among the 17 reference strains based on the method of Oliphant *et al*. with some modifications [22]. The phylogenetic tree was constructed by BIOTA Inc. (Tokyo, Japan). In detail, this pipeline used Prodigal [23] and HMMER [24] to identify 81 core bacterial genes, MAFFT [25] to perform multiple sequence alignments of the genes, and FastTree2 [26] to perform phylogeny reconstruction. Instead of calculating the bootstrap values, this pipeline calculates the gene support index (GSI), which indicates the number of gene trees that support each branch in the resulting tree obtained from the concatenated sequence. The phylogenetic tree was constructed using FigTree v1.4.4, available at http://tree.bio.ed.ac.uk/software/figtree/.

### Average nucleotide identity (ANI), in-silico DNA-DNA hybridization (DDH), amino acid identity (AAI), and amino acid identity of conserved genes (cAAI)

ANI was calculated using the ANI calculator (http://enve-omics.ce.gatech.edu/ani/) [27]; digital DDH (dDDH) values of strain WR16-4^T^ and its phylogenetically closest neighbors were estimated using the Genome-to-Genome Distance Calculator (GGDC 3.0; https://ggdc.dsmz.de/ggdc.php) with recommended Formula 2 [28]; AAI values were calculated using the AAI calculator (http://enve-omics.ce.gatech.edu/aai/) [29]; and cAAI analysis was performed by BIOTA Inc. To minimize the impact of horizontal gene transfer (HGT) on the phylogenetic relationship, AAI values were calculated using only orthologous genes. Therefore, cAAI was calculated between each pair of orthologous genes of 17 strain genome sequences using CompareM (https://github.com/donovan-h-parks/CompareM).

## Results

### Growth of strain WR16-4^T^ on solid media

**Fig 1:**
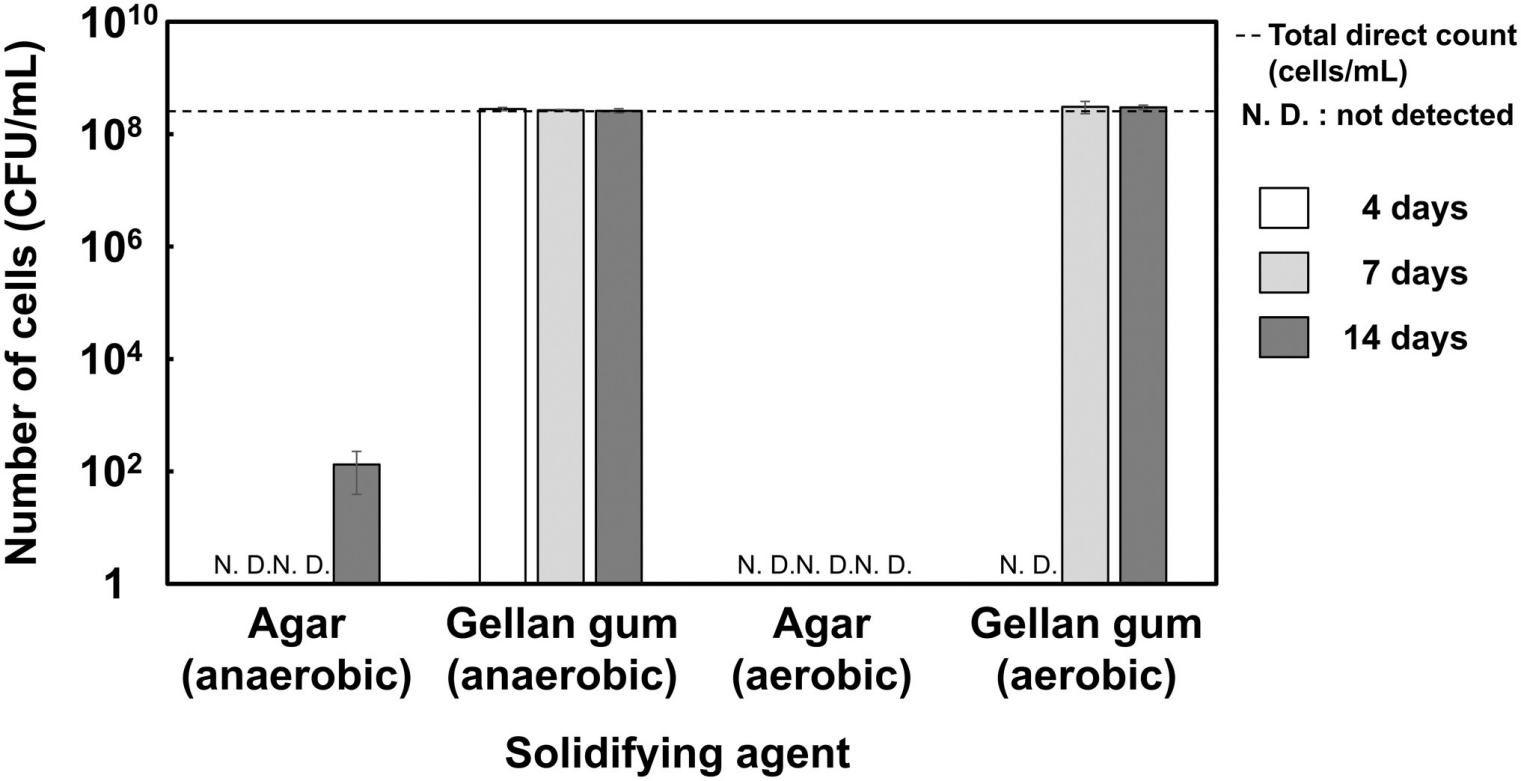
Effect of media solidifying agents on strain WR16-4^T^ colony formation. Data are expressed as mean ± SD (n = 3). N. D., not detected.

Preliminary experiments suggested that the growth of strain WR16-4^T^ was suppressed on agar compared to that in liquid medium. Therefore, the colony-forming ability of strain WR16-4^T^ on the plate was compared using gellan gum and agar as solidifying agents (Fig 1 and S3 Table). Viable cells formed colonies on the gellan gum plate after 4 d incubation under anaerobic conditions, with an average colony size of approximately 1, 2–3, and 3–4 mm, after 4, 7, and 14 d, respectively. Under aerobic conditions, no colonies were observed on the gellan gum plate at 4 d whereas small colonies (approximately 0.5 mm) formed after 7 d in a similar number as that under anaerobic conditions. In contrast, no colonies formed on agar media under aerobic conditions even after 14 d. Although colonies appeared under anaerobic conditions after 14 d, the number of colonies was 10^6^-fold lower than the total number of inoculated bacteria.

### Carbohydrate fermentation profile

Strain WR16-4^T^ exhibited unique characteristics; it slowly grew on a normal MRS medium and was difficult to culture in the absence of fermented vegetable extracts. Therefore, carbohydrate fermentation tests were performed using the API 50CH system to identify preferable carbon sources to improve cell growth. Growth was evaluated on API 50 CHL medium (pH 6.7) and a modified medium prepared with the same components but at pH 5.0. Positive reactions were recorded for d-fructose and sucrose in both these media. In addition, a weakly positive reaction was recorded for d-turanose, gluconate, and 5-ketogluconate only when using the modified medium. All other carbohydrates were not utilized.

### Physiological, morphological, and biochemical characteristics

**Fig 2:**
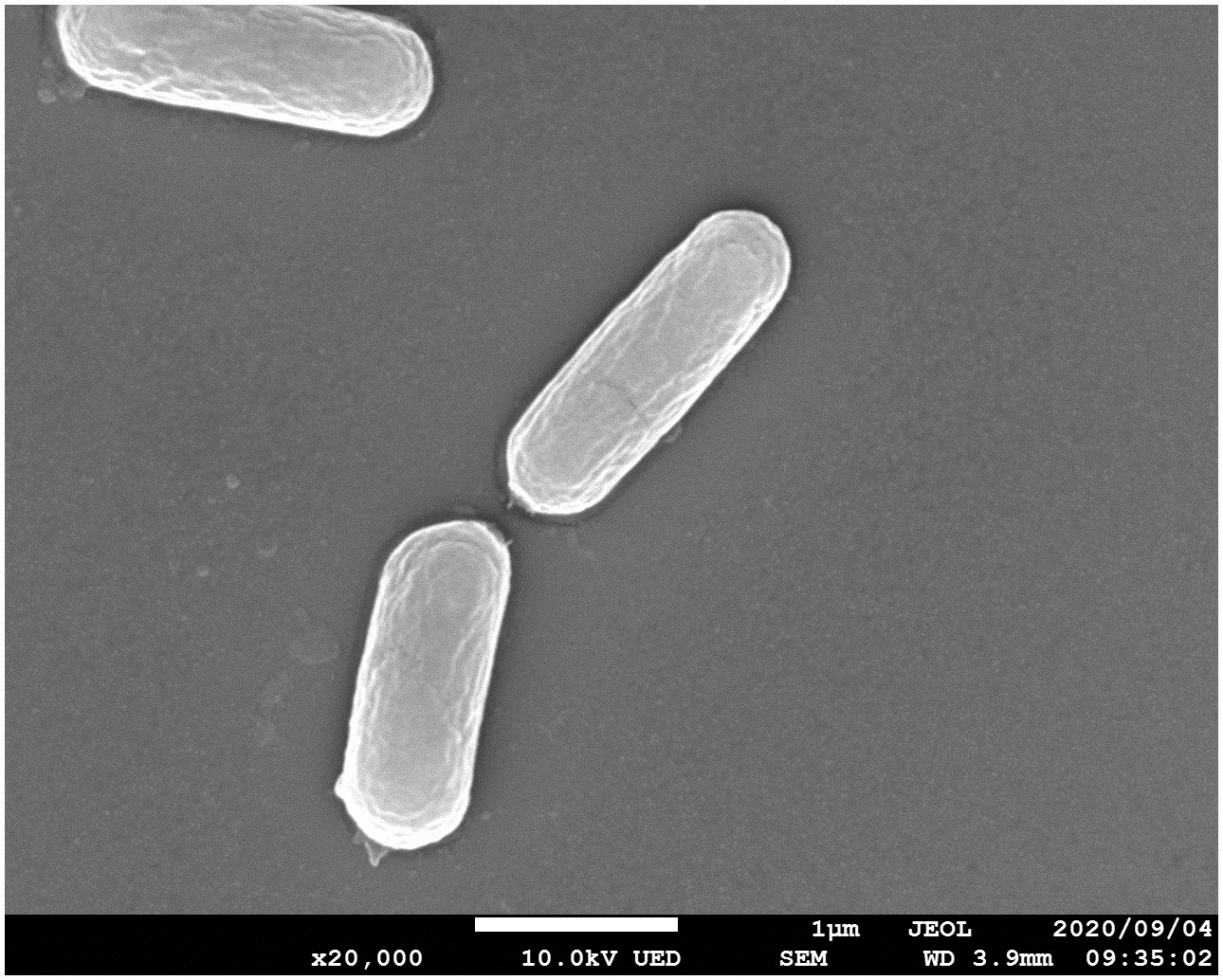
Scanning electron microscopy (SEM) images of strain WR16-4^T^.

We then investigated the physiological, morphological, and biochemical characteristics of strain WR16-4^T^. The cells were identified by SEM analyses as straight and rod-shaped, with a size of 1.5–2.5 μm × 0.5–0.7 μm (Fig 2). The cells were found to be catalase-negative, Gram-stain-positive, non-motile, and non-spore-forming. No growth occurred on 20% (w/v) d-glucose as a carbon source under anaerobic and aerobic conditions (Table 1). The addition of 0.5% (w/v) pyruvate to the medium did not improve the cell growth under both aeration conditions. In contrast, the addition of 0.5% (w/v) d-fructose to the medium promoted good cell growth under both conditions at 48 h. Gas was produced from sucrose or d-glucose with 0.5% (w/v) d-fructose. Cell growth was observed on 20% (w/v) sucrose at 30°C, but not at 15°C or 45°C during 48 h, and slow growth occurred at 15°C until 120 h. No growth occurred in the presence of 2% (w/v) NaCl. Total soluble sugars (16.6 g), including d-glucose (13.1 g), were consumed, producing lactic acid (4.3 g) and acetic acid (0.2 g). Any other organic acids were not detected. The major cellular fatty acid was C_16:0_ (53.87%), followed by C_19_ cycloprop. 11,12 (30.42%). Other minor components were C_18:1_ ω9c (5.39%), C_18:0_ (1.36%), C_16:1_ ω7c (1.28%), C_14:0_ (1.20%), C_12:0_ (0.30%), C_10:0_ (0.22%), C_16:0_ 3OH (0.14%), and C_19:0_ cycloprop. 11,12 DMA (0.09%). The cell wall peptidoglycan contained aspartic acid, glutamic acid, lysine, and alanine (type A4α (l-Lys-d-Asp)).

**Table 1:** Phenotypic characteristics of strain WR16-4^T^.

Substrate (20% (w/v))	Temperature (°C)	Cultivation time		Aeration		Additive (0.5% (w/v))		NaCl (2% (w/v))	Growth
		48 (h)	120 (h)	Anaerobic	Aerobic	Pyruvate	d-fructose		
d-glucose	30	✓		✓					–
	30		✓	✓					–
	30	✓			✓				–
	30		✓		✓				–
	30	✓		✓		✓			–
	30		✓	✓		✓			–
	30	✓			✓	✓			–
	30		✓		✓	✓			–
	30	✓		✓			✓		+
	30	✓			✓		✓		+
Sucrose	15	✓		✓					–
	15		✓	✓					+
	30	✓		✓					+
	30	✓		✓				✓	–
	45	✓		✓					–
	45		✓	✓					–

+, positive; −, negative

### Optimization of strain WR16-4^T^ growth conditions

**Fig 3:**
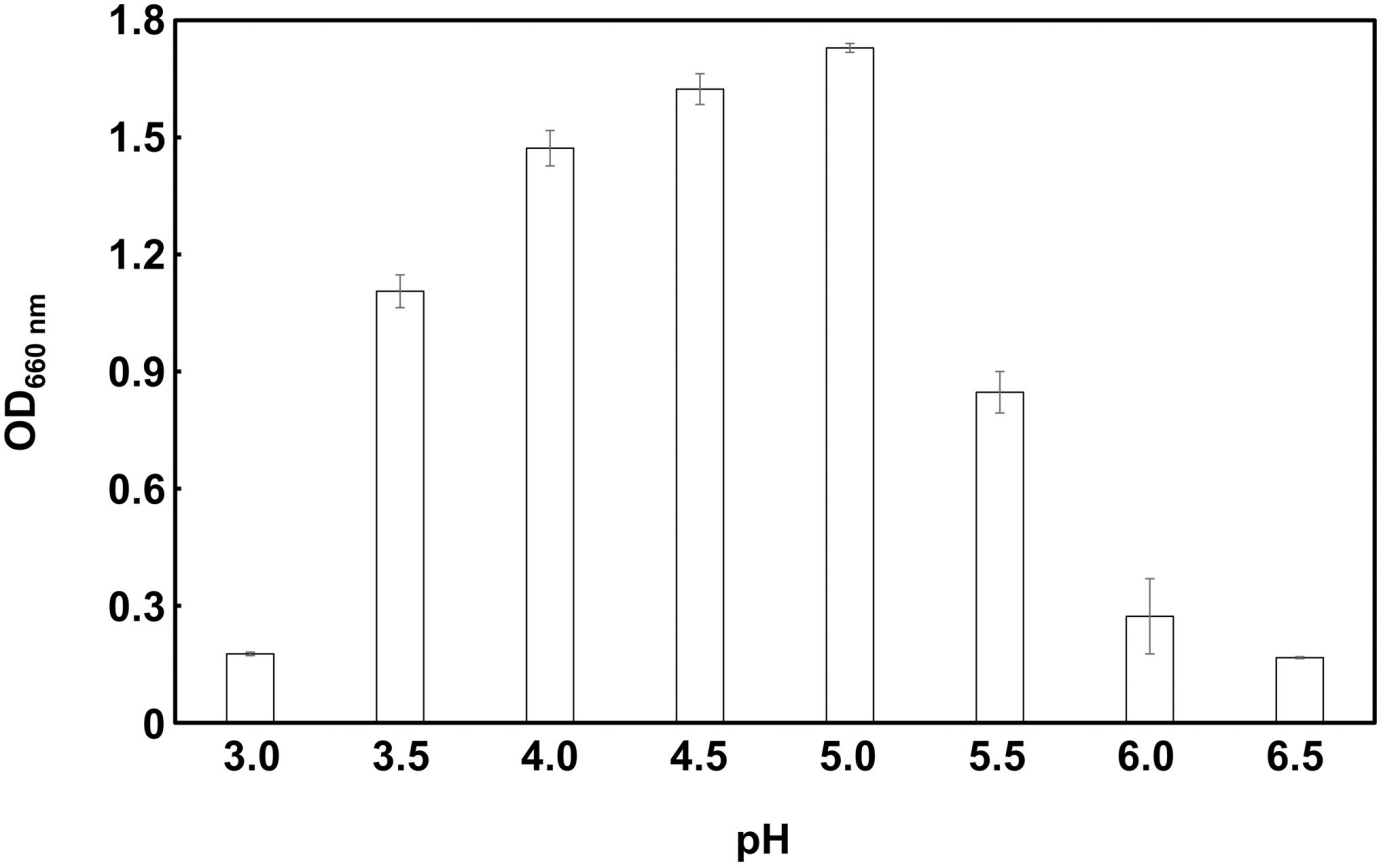
Assessment of optimal pH for the growth of strain WR16-4^T^. OD_660 nm_: optical density at 660 nm. Data are expressed as mean ± SD (n = 3).

The optimal pH and sucrose concentration for strain WR16-4^T^ were investigated to optimize the growth conditions (Fig 3 and S4 Table). Maximum growth was observed at pH 4.5–5.0, with slightly reduced growth at pH 3.5, 4.0, and 5.5. However, the cell growth was considerably suppressed at pH 6.0–6.5 and pH 3.0.

**Fig 4:**
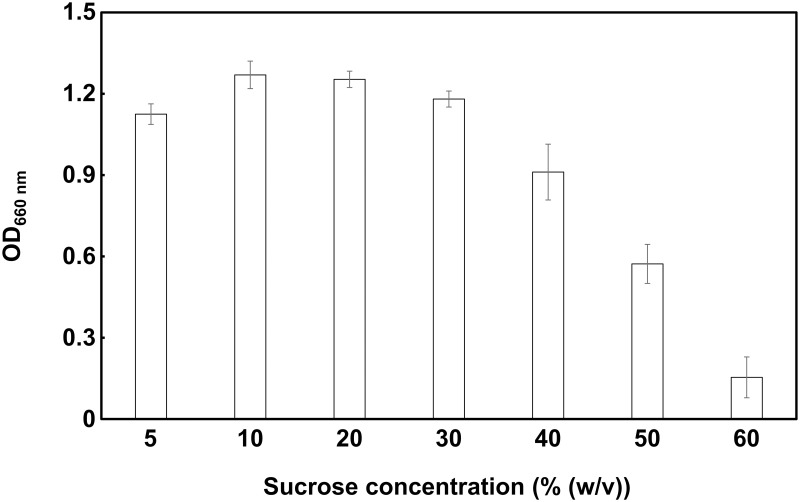
Influence of sucrose concentration on the growth of strain WR16-4^T^. OD_660 nm_: optical density at 660 nm. Data are expressed as mean ± SD (n = 3).

The growth of strain WR16-4^T^ in a modified MRS medium (pH 4.5) with high sucrose concentration is shown in Fig 4 and S5 Table. Maximum growth was observed on 5–30% (w/v) sucrose, especially at 10% (w/v) (OD_660 nm_ = 1.27) and 20% (w/v) (OD_660 nm_ = 1.25) concentrations. Notably, this bacterium was able to grow on high sucrose concentrations up to 50% (w/v) and showed weak growth even in 60% (w/v) sucrose.

**Fig 5:**
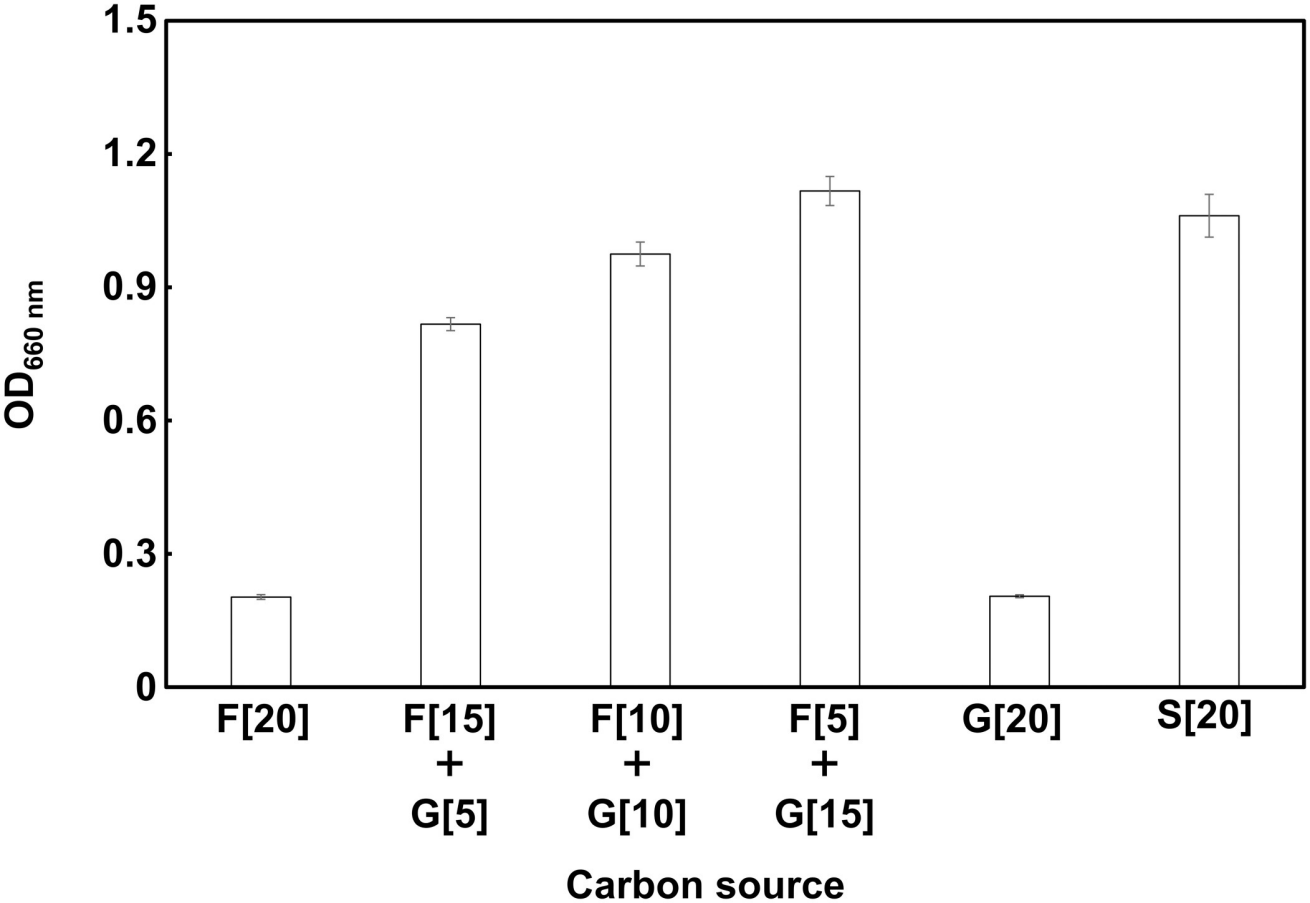
Effect of carbon source combinations on the growth of strain WR16-4^T^. F: d-fructose, G: d-glucose, S: sucrose; numbers in square brackets denote % (w/v) of carbon source added. OD_660 nm_: optical density at 660 nm. Data are expressed as mean ± SD (n = 3).

The effect of the presence of d-glucose and/or d-fructose, which are the constituents of sucrose, in the medium on the growth of strain WR16-4^T^ was evaluated to clarify the carbon source requirement (Fig 5 and S6 Table). The bacterial growth was significantly reduced with solely d-glucose or d-fructose as a carbon source. In contrast, the medium supplemented with both d-glucose and d-fructose had a beneficial effect on cell growth, despite the high total carbohydrate concentration. In particular, the turbidity under F[10]+G[10] or F[5]+G[15] conditions was similar to or higher than that of the positive control containing 20% (w/v) sucrose as a carbon source (S[20]).

### Genome and phylogenetic analysis

**Fig 6:**
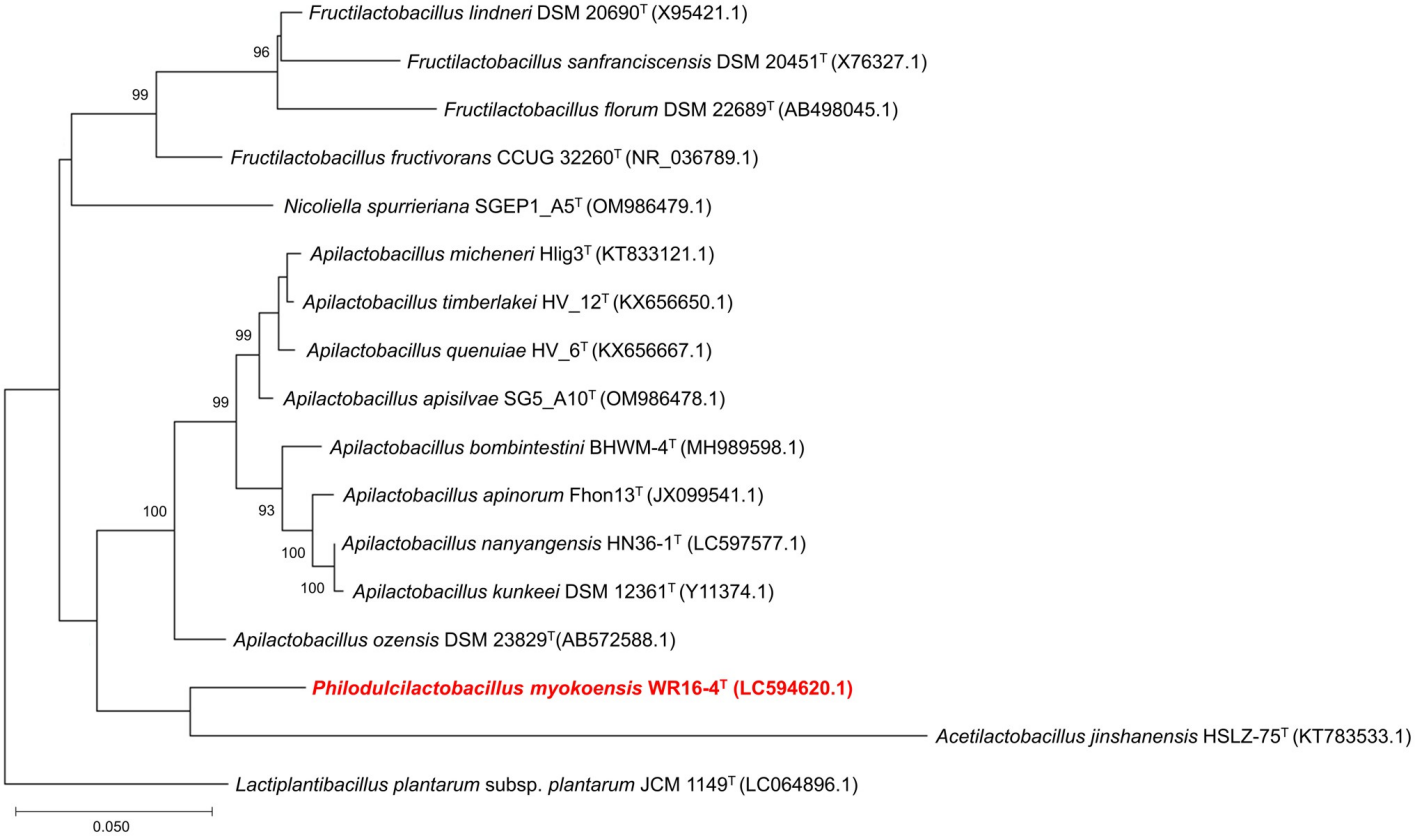
Phylogenetic tree of the strain WR16-4^T^ derived from 16S rRNA gene sequence analyses using the maximum-likelihood method. *L*. *plantarum* subsp. *plantarum* JCM 1149^T^ was used as the outgroup. Numbers at nodes denote the bootstrap values (>90%) for branch points based on 1,000 replications.

The 16S rRNA gene sequence of strain WR16-4^T^ obtained was 1,494 bp. The 16S rRNA gene sequence analysis revealed that strain WR16-4^T^ was related to the *Apilactobacillus* and *Lentilactobacillus* genera. The closest relatives were the type strains of *A*. *ozensis* DSM 23829^T^ (93.1%) and *Lentilactobacillus parafarraginis* JCM 14109^T^ (93.1%), and *Lentilactobacillus kefiri* JCM 5818^T^ was the next closest relative (92.9%). Furthermore, the sequence similarities were also compared to other FLAB strains, including *F*. *fructivorans* KCTC 3543^T^ (92.3%), *N*. *spurrieriana* SGEP1_A5^T^ (91.8%), and *A*. *jinshanensis* HSLZ-75^T^ (90.0%). However, the sequence similarities were <94.5%, below the limit threshold for the correct genus-level assignment [30]. Phylogenetic analysis of 16S rRNA sequences using the maximum-likelihood method showed that this novel strain was most closely related to the type strain of *A*. *jinshanensis* HSLZ-75^T^ (Fig 6).

**Fig 7:**
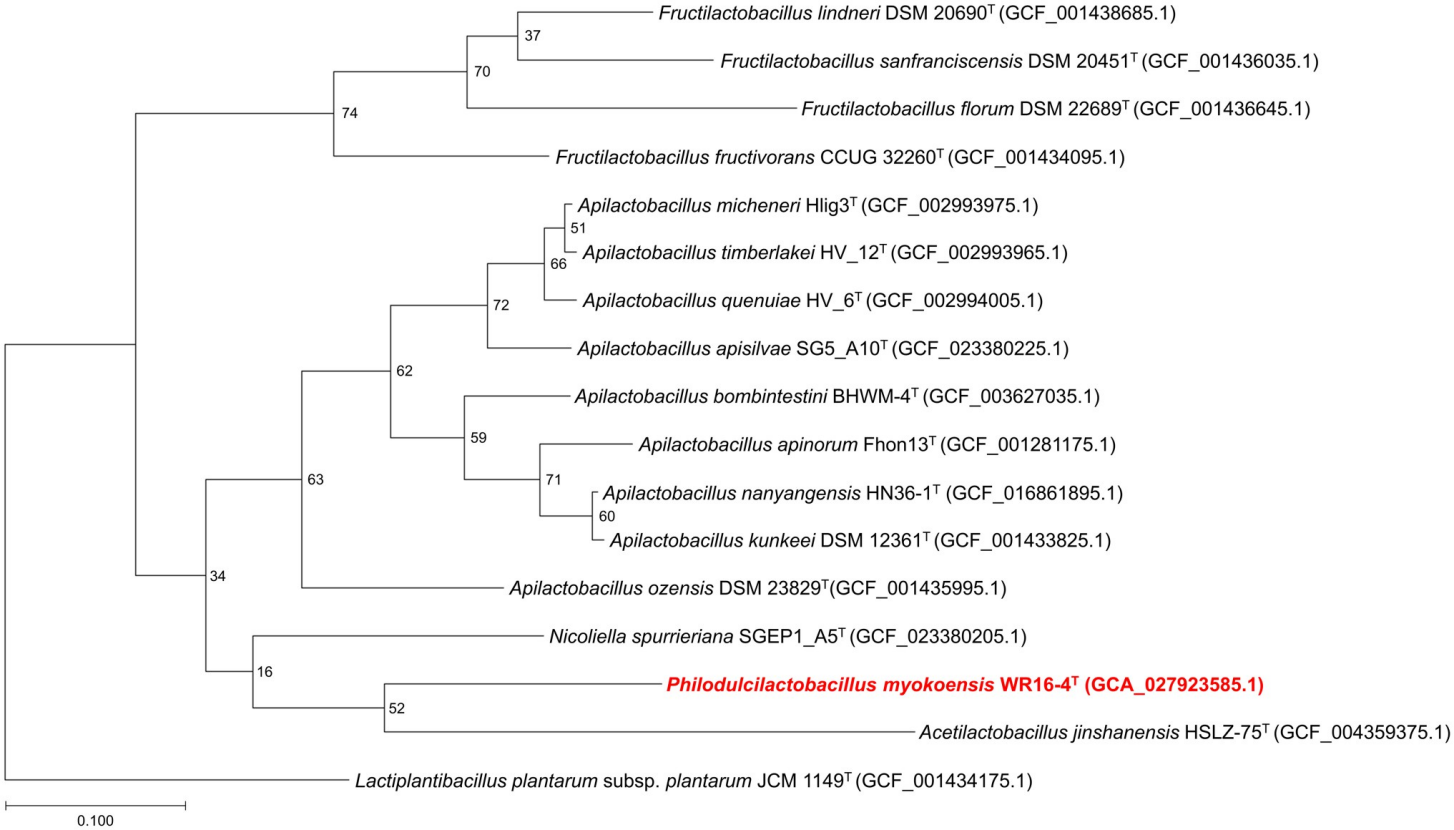
Core genome phylogenetic tree obtained from the concatenated multiple sequence alignments of 81 core bacterial genes using UBCG2. *L*. *plantarum* subsp. *plantarum* JCM 1149^T^ was used as an outgroup to root the tree. Bipartitions of the phylogenetic tree show the gene support index (GSI) values calculated by UBCG2 of ≥ 95%. The gene support index (GSI) is indicated at branching points in the concatenated phylogenetic tree. The horizontal bar at the base represents 0.10 substitutions per nucleotide site.

The total length of the genome of strain WR16-4^T^ was 1,672,155 bp with a total of 1,611 protein-coding genes (CDS), 15 rRNA genes, 63 tRNA genes, and 33.5% G+C content. The core genome phylogenetic tree suggested that this novel strain was most closely related to the type strain of *A*. *jinshanensis* HSLZ-75^T^ (Fig 7). The ANI, dDDH, AAI, and cAAI values of strain WR16-4^T^ were compared to those of the closest type strains (genus: *Acetilactobacillus*, *Nicoliella*, *Apilactobacillus*, *Fructilatcobacillus*, and *Lactiplantibacillus* [outgroup]) based on the phylogenetic tree reconstructed in this study (Table 2 and S7 Table). The ANI values ranged from 73.36 to 78.28% (except for the outgroup), which were below the species cut-off value (95%) [31]. The dDDH values between strain WR16-4^T^ and type strains were 16.3 and 32.9% (except for the outgroup), respectively, which were significantly lower than the 70% threshold for species demarcation [32]. The AAI values ranged from 53.96 to 60.88% (except for the outgroup), which were significantly below the threshold for genus demarcation (68%) [29]. The highest cAAI values compared to those of WR16-4^T^ were those of the genus *Apilactobacillus*, with values ranging from 62.51 to 63.79%. The cAAI values between strain WR16-4^T^ and *N*. *spurrieriana* SGEP1_A5^T^ and *A*. *jinshanensis* HSLZ-75^T^, reached 62.87% and 62.03%, respectively. The lowest cAAI values were observed when comparing strain WR16-4^T^ with genus *Fructilactobacillus*, with values ranging from 58.00 to 61.04%.

**Table 2:** Genotypic characteristics of strain WR16-4^T^ and closely related species.

Species	Strain	Accession No.	ANI (%)	dDDH (%)	AAI (%)	cAAI (%)
*Philodulcilactobacillus myokoensis*	WR16-4^T^	GCA_027923585.1	100	100	100	100
*Acetilactobacillus jinshanensis*	HSLZ-75^T^	GCF_004359375.1	78.18	20.3	58.86	62.03
*Nicoliella spurrieriana*	SGEP1_A5^T^	GCF_023380205.1	77.19	22.3	60.31	62.87
*Apilactobacillus ozensis*	DSM 23829^T^	GCF_001435995.1	76.03	18.0	60.32	63.12
*Apilactobacillus apinorum*	Fhon13^T^	GCF_001281175.1	75.71	18.1	59.57	62.51
*Apilactobacillus apisilvae*	SG5_A10^T^	GCF_023380225.1	78.05	18.8	60.78	63.44
*Apilactobacillus bombintestini*	BHWM-4^T^	GCF_003627035.1	78.28	19.4	60.68	63.79
*Apilactobacillus kunkeei*	DSM 12361^T^	GCF_001433825.1	76.29	19.4	59.79	62.57
*Apilactobacillus micheneri*	Hlig3^T^	GCF_002993975.1	76.28	18.5	60.77	63.67
*Apilactobacillus nanyangensis*	HN36-1^T^	GCF_016861895.1	76.33	18.4	59.67	62.57
*Apilactobacillus quenuiae*	HV_6^T^	GCF_002994005.1	75.68	17.6	60.88	63.48
*Apilactobacillus timberlakei*	HV_12^T^	GCF_002993965.1	75.83	18.0	60.55	63.48
*Fructilactobacillus florum*	DSM 22689^T^	GCF_001436645.1	N. A.	32.9	53.96	58.00
*Fructilactobacillus fructivorans*	CCUG 32260^T^	GCF_001434095.1	75.46	19.9	57.22	61.04
*Fructilactobacillus lindneri*	DSM 20690^T^	GCF_001438685.1	74.90	21.5	55.61	59.43
*Fructilactobacillus sanfranciscensis*	DSM 20451^T^	GCF_001436035.1	73.36	16.3	56.10	59.51
*Lactiplantibacillus plantarum* subsp. *plantarum*	JCM 1149^T^	GCF_001434175.1	77.26	27.6	52.92	57.80

ANI, average nucleotide identity; DDH, in-silico DNA-DNA hybridization; AAI, amino acid identity; cAAI, amino acid identity of conserved genes; N. A. not available (the ANI values are too close to the detection limit.)

The criteria to define new genera in the taxonomic reclassification of lactic acid bacteria are: (i) genera are monophyletic, (ii) intra- and inter-genus pairwise AAI/cAAI values show minimal overlap, and (iii) genera are differentiated by characteristic metabolic or ecological traits [33, 34]. The inter- and intra-genus cAAI values are generally lower and higher than 68%, respectively, with a transition zone of 65 to 71% in the *Lactobacillaceae* [33, 34]. In summary, the ANI and dDDH values of strain WR16-4^T^ compare to those of the analyzed type strains were below the species limit thresholds, and the AAI and cAAI values of strain WR16-4^T^ were lower than the genus threshold. Furthermore, the 16S rRNA phylogenetic tree and core genome phylogenetic tree revealed strain WR16-4^T^ as a sister clade to *Apilactobacillus* and the most closely related genus was *Acetilactobacillus*. Overall, strain WR16-4^T^ was determined to be a novel genus.

### Description of *Philodulcilactobacillus* gen. nov.

*Philodulcilactobacillus*: Phi.lo.dul.ci.lac.to.ba.cil’lus. N.L. masc. adj. philus -a -um, friend, loving; from Gr. masc. adj. philos -ê -on, loving; L. masc./fem. adj. dulcis, sweet; N.L. masc. n. *Lactobacillus* a bacterial genus; *Philodulcilactobacillus*, a lactobacillus that can grow under high sugar concentrations. The bacterial cells are Gram-stain-positive, non-spore-forming, non-motile, rods, and catalase-negative. The major cellular fatty acids are C_16:0_ and C_19_ cycloprop. The type strain G+C content is 33.5%. The genus is a member of the family *Lactobacillaceae* of the order *Lactobacillales* of the class *Bacilli* of the phylum *Bacillota*. The type species is *Philodulcilactobacillus myokoensis*.

### Description of *Philodulcilactobacillus myokoensis* sp. nov.

The proposed novel strain is *Philodulcilactobacillus myokoensis* sp. nov. (myo.ko.en’sis. N.L. masc. adj. *myokoensis*, pertaining to Myoko, a city in the Niigata Prefecture of Japan, from where the first strain was isolated). Cells are Gram-stain-positive, non-spore-forming, non-motile, rods, and catalase-negative, with a size of 1.5–2.5 μm × 0.5–0.7 μm. Grows on gellan gum plates with 20% (w/v) sucrose but not on d-glucose or agar plates. White and circular colonies are formed in an anaerobic environment on an MRS medium (pH 4.5) with 20% (w/v) sucrose instead of d-glucose, and gellan gum instead of agar. Ferments d-fructose and sucrose and weakly ferments d-turanose, gluconate, and 5-ketogluconate at pH 5.0. Grows well at pH 3.5–5.5, with optimal growth at pH 4.5–5.0 and weak growth at pH 3.5, 4.0, and 5.5. Grows between 15–30°C, with optimal growth at 30°C and no growth at 45°C. Produces lactic acid, a small amount of acetic acid, and gas in the presence of both d-glucose with d-fructose. Produces gas from sucrose. The cell wall murein is type A4α (l-Lys-d-asp), and the major cellular fatty acids are C_16:0_ and C_19_ cycloprop. 11,12. The G+C content of genomic DNA is 33.5%. Cells are culturable even at high sucrose concentrations, with optimal growth at 10–20% (w/v) and can grow on up to 50% (w/v) sucrose.

The type strain is WR16-4^T^ (= NBRC 115064^T^ = DSM 112857^T^) isolated from fermented vegetable extracts collected from an industry located in Myoko City, Niigata Prefecture, Japan. The DDBJ/GenBank/EMBL accession number for the 16S rRNA gene sequence of strain WR16-4^T^ is LC594620.1 and that of the draft genome sequence is GCA_027923585.1 (BioProject number PRJDB13692).

## Discussion

The genus *Lactobacillus* comprises 261 species (as of March 2020) that are extremely diverse at the phenotypic, ecological, and genotypic levels. Zheng *et al*. proposed the reclassification of the genus *Lactobacillus* into 25 genera based on a polyphasic approach [33]. Various media (MRS, BCP, TYP, M17, etc.) have been used for the culture and isolation of these general lactic acid bacteria; however, glucose is used as the primary carbon source as it is the most common substrate to study heterotrophic metabolism [35, 36]. Furthermore, the pH values of these media are adjusted to near neutral as most bacteria, including lactic acid bacteria, grow well under these conditions. In this study, we achieved the isolation of a novel lactic acid bacterium that is difficult to culture with d-glucose as the only carbon source but grows easily with sucrose as a carbon source under acidic conditions.

The API 50 CH test indicated that strain WR16-4^T^ can consume a few carbohydrates, especially preferring sucrose or d-fructose but not d-glucose as a carbon source. In addition, strain WR16-4^T^ had a high sucrose fermentation potential, and the carbohydrate combination test revealed that growth on d-fructose alone was slower than that on sucrose. These results suggest that the isolated strain only consumed fructose from the API 50 CHL medium but not from the MRS medium, possibly owing to the difference in media composition and/or potential suppression of cell growth at higher initial d-fructose concentrations. Endo *et al*. have described that the FLAB *Apilactobacillus* (reclassification from *Lactobacillus* sp.), *Fructilactobacillus* (reclassification from *Lactobacillus* sp.), and *Fructobacillus*, exhibit poor growth on glucose in the absence of electron acceptors, such as oxygen, pyruvate, or fructose [37]. Strain WR16-4^T^ exhibited similar characteristics with no growth on d-glucose under normal conditions; however, it grew only when d-fructose was added to the MRS medium and not on d-glucose in the presence of oxygen and/or pyruvate. This result suggests that the new strain may not be able to utilize oxygen and/or pyruvate as an electron acceptor. Physiological, biochemical, genomic, and phylogenetic analyses indicated that strain WR16-4^T^ is close to the species in the genera *Acetilactobacillus*, *Nicoliella*, and *Apilactobacillus* (Table 3). *A*. *jinshanensis* has some unique characteristics, such as optimum growth at pH 4.0, utilization of sugar alcohols and disaccharides as sole carbon sources but most hexoses and all pentoses are not, and produced dextran from sucrose [33, 38]. *N*. *spurrieriana* is facultatively anaerobic and obligately fructophilic, and its growth on d-glucose is enhanced with pyruvate, d-fructose and, to a minimal extent, oxygen [22]. *Apilactobacillus* sp. is rod-shaped, heterofermentative, grows at 15–37°C, and has a DNA G+C content ranging from 30.5 to 36.4 mol%; many strains grow under acidic conditions below pH 3.0, and all strains in the genus convert fructose to mannitol [33]. *A*. *micheneri* Hlig3^T^, *A*. *quenuiae* HV_6^T^, and *A*. *timberlakei* HV_12^T^ grow well on MRS medium containing 20% d-fructose under aerobic conditions [40]. *A*. *ozensis* DSM 23829^T^ forms colonies on MRS agar under anaerobic conditions but not under aerobic conditions [42]. *A*. *kosoi* NBRC 113063 (heterotypic synonym of *A*. *micheneri*), obtained from sugar-vegetable fermented beverage by Chiou *et al*., exhibits increased growth with the addition of d-fructose (5–20%) to the MRS medium but not with the addition of 0.5% pyruvic acid. In addition, the strain was culturable at pH 4.0–7.0 (optimum pH 6.5) and required an MRS agar containing 5–10% d-fructose for colony formation [43, 44]. *A*. *apisilvae* SG5_A10^T^ is facultatively anaerobic and obligately fructophilic, and the growth in d-glucose is enhanced with pyruvate, d-fructose and, to a minimal extent, oxygen [22]. Strain WR16-4^T^ exhibited some properties similar to those of the members in these genera, such as a requirement for a specific electron acceptor to use d-glucose as a carbon source, growth under acidic conditions, and a heterofermentative and fructophilic nature. However, the strain also has several unique characteristics; it does not grow well on agar medium, does not effectively utilize oxygen and/or pyruvate as an electron acceptor, has an optimum pH of 4.5–5.0 and does not grow well at a near neutral pH, and exhibits resistance to high sucrose concentrations.

**Table 3:** Comparison of the characteristics of strain WR16-4^T^ and closely related species.

Characteristic	1	2	3	4	5	6	7	8	9	10	11	12
Temperature range (°C)	15–30	20–40	17–37	17–30	15–50	15–35	15–35	15–35	15–37	15–37	20–37	15–37
pH range	3.5–5.5	3.0–5.0	4.0–7.0	4.5–7.0	3.0–12.0	2.0–6.0	2.0–6.0	2.0–7.0	3.7–8.0	N. A.	2.0–8.0	5.0
Utilization of												
Glucose	–	+	+	+	+	+	+	+	+	+	+	+
Fructose	+	–	+	+	+	+	+	+	+	+	+	+
Sucrose	+	–	+	+	–	–	–	+	+	w	–	+
Reference	This study	[38]	[22]	[22]	[39]	[40]	[40]	[40]	[41]	[42]	[45, 46]	[47]

Strains:1, *P*. *myokoensis* WR16-4^T^; 2, *A*. *jinshanensis* HSLZ-75^T^; 3, *N*. *spurrieriana* SGEP1_A5^T^; 4, *A*. *apisilvae* SG5_A10^T^; 5, *A*. *apinorum* Fhon13^T^;

6 *A*. *micheneri* Hlig3^T^; 7, *A*. *timberlakei* HV_12^T^; 8, *A*. *quenuiae* HV_6^T^; 9, *A*. *kunkeei* DSM 12361^T^; 10, *A*. *ozensis* DSM 23829^T^;

11, *A*. *bombintestini* BHWM-4^T^; 12, *A*. *nanyangensis* HN36-1^T^

+ positive;–negative; w weak positive; N.A. not available.

Several studies have reported that the use of gellan gum instead of agar in solid media improves the growth of slow-growing bacteria [48–50]. Hara *et al*. found that agar contains 5-hydroxymethylfuran-2-carboxylic acid and furan-2-carboxylic acid, which inhibit the proliferation of some slow-growing or difficult-to-culture bacteria on the plate [51]. The presence of these and/or other unknown inhibitory components might account for the suppression of WR16-4^T^ growth on solid media. In addition, this strain formed colonies on both solid media faster under anaerobic conditions than under aerobic conditions. These results suggest that oxygen is not only difficult to be used as an electron acceptor, as described, but it also causes some stress for the isolated strain on a solid medium.

In summary, the present study successfully isolated and identified the bacterial strain WR16-4^T^ from fermented vegetable extracts, proposing its classification as a new species, *Philodulcilactobacillus myokoensis*, based on its physiological, morphological, and phenotypical characteristics. The type strain is WR16-4^T^ (= NBRC 115064^T^ = DSM 112857^T^). This study provides a comprehensive description of the characteristics of the novel strain, especially its growth on sucrose. Future studies should further analyze the strain to better understand its metabolic properties and potential implications in food safety and food processing.

## Supporting information

S1 Table Component of fermented vegetable extract. (PDF)

S2 Table Component of boiled wild grasses. (PDF)

S3 Table Data for Fig 1. (PDF)

S4 Table Data for Fig 3. (PDF)

S5 Table Data for Fig 4. (PDF)

S6 Table Data for Fig 5. (PDF)

S7 Table The pairwise amino acid identity of conserved genes (cAAI). (PDF)
