# Supplementary material for: Philodulcilactobacillus myokoensis gen. nov., sp. nov., a fructophilic, acidophilic, and agar-phobic lactic acid bacterium isolated from fermented vegetable extracts
Source: PLoS One. 2023 Jun 21;18(6):e0286677. doi: 10.1371/journal.pone.0286677 (PMC10284405; doi:10.1371/journal.pone.0286677)
Supplement: S1 Table — (PDF) [file pone.0286677.s001.pdf]

S1 Table. Component of fermented vegetable extract.

Fermented extract	L
Bean sprouts fee	6.42
Cucumber	4.39
Spinach	1.15
Cabbage	7.83
Onion	2.34
Parsley	0.36
Potato	1.45
Carrot	1.59
Broccoli	4.00
Burdock	1.28
Lotus root	1.31
Japanese mustard spinach	0.14
Turnip	0.08
Tomato	0.41
Japanese white radish	2.28
Pineapple	2.03
Apple	1.60
Lemon	0.34
Aloe	1.00
Kelp	1.00
