# Supplementary material for: Philodulcilactobacillus myokoensis gen. nov., sp. nov., a fructophilic, acidophilic, and agar-phobic lactic acid bacterium isolated from fermented vegetable extracts
Source: PLoS One. 2023 Jun 21;18(6):e0286677. doi: 10.1371/journal.pone.0286677 (PMC10284405; doi:10.1371/journal.pone.0286677)
Supplement: S2 Table — (PDF) [file pone.0286677.s002.pdf]

S2 Table. Component of boiled wild grasses.

Material	kg/600 L
Fish Mint	10.000
Plantain	6.500
Job's tears	6.000
Java-bean	4.000
Mugwort	4.000
Field horsetail	3.500
Chenopod	3.200
Ginkgo leaves	2.000
Glycyrrhiza	2.000
Honeysuckle	2.000
Silver vine	2.000
Boxthorn leaves	1.500
New Zealand spinach	1.500
Jiaogulan	1.000
Turmeric	1.000
Siberian ginseng	1.000
SuperHerb	1.000
Japanese Grand Ivy	1.000
Cassia	1.000
Dayflower	1.000
Coffee senna	1.000
pine needle	1.000
Shiitake mushroom	1.000
Japanese yam	1.000
Maitake mushroom	0.950
Jack bean	0.620
Mallotus bark	0.500

Fiveleaf aralia	0.500
Kuma bamboo gras	0.500
Dandelion	0.500
Heavenly bamboo leaves	0.500
Tsuchi-akebi	0.005
