# Supplementary material for: Philodulcilactobacillus myokoensis gen. nov., sp. nov., a fructophilic, acidophilic, and agar-phobic lactic acid bacterium isolated from fermented vegetable extracts
Source: PLoS One. 2023 Jun 21;18(6):e0286677. doi: 10.1371/journal.pone.0286677 (PMC10284405; doi:10.1371/journal.pone.0286677)
Supplement: S3 Table — (PDF) [file pone.0286677.s003.pdf]

S3 Table. Data for Fig 1.

			CFU/mL (1)	CFU/mL (2)	CFU/mL (3)	CFU/mL (Ave.)	SD
Gellan gum	Aerobic	4 days	N. D.	N. D.	N. D.	N. D.	N. D.
		7 days	300000000	340000000	280000000	306666666.7	30550504.63
		14 days	320000000	320000000	260000000	300000000	34641016.15
	Anaerobic	4 days	256000000	302000000	282000000	280000000	23065125.19
		7 days	268000000	276000000	256000000	266666666.7	10066445.91
		14 days	254000000	292000000	236000000	260666666.7	28589042.19
Agar	Aerobic	4 days	N. D.	N. D.	N. D.	N. D.	N. D.
		7 days	N. D.	N. D.	N. D.	N. D.	N. D.
		14 days	N. D.	N. D.	N. D.	N. D.	N. D.
	Anaerobic	4 days	N. D.	N. D.	N. D.	N. D.	N. D.
		7 days	N. D.	N. D.	N. D.	N. D.	N. D.
		14 days	200	200	0	133.3333333	115.4700538
