# Supplementary material for: Philodulcilactobacillus myokoensis gen. nov., sp. nov., a fructophilic, acidophilic, and agar-phobic lactic acid bacterium isolated from fermented vegetable extracts
Source: PLoS One. 2023 Jun 21;18(6):e0286677. doi: 10.1371/journal.pone.0286677 (PMC10284405; doi:10.1371/journal.pone.0286677)
Supplement: S4 Table — (PDF) [file pone.0286677.s004.pdf]

S4 Table. Data for Fig 3.

pH	$OD_{660\ nm}$ (1)	$OD_{660\ nm}$ (2)	$OD_{660\ nm}$ (3)	$OD_{660\ nm}$ (Ave.)	SD
3.0	0.1719	0.1804	0.1778	0.1767	0.004355456
3.5	1.0574	1.1262	1.1335	1.1057	0.041987974
4.0	1.4536	1.4394	1.5243	1.472433333	0.045475525
4.5	1.5992	1.6028	1.6692	1.623733333	0.039416409
5.0	1.725	1.7205	1.7422	1.729233333	0.011452656
5.5	0.8787	0.7857	0.8767	0.847033333	0.053125637
6.0	0.2055	0.3832	0.23	0.2729	0.09630488
6.5	0.1666	0.1646	0.1694	0.166866667	0.002411086
