# Supplementary material for: Philodulcilactobacillus myokoensis gen. nov., sp. nov., a fructophilic, acidophilic, and agar-phobic lactic acid bacterium isolated from fermented vegetable extracts
Source: PLoS One. 2023 Jun 21;18(6):e0286677. doi: 10.1371/journal.pone.0286677 (PMC10284405; doi:10.1371/journal.pone.0286677)
Supplement: S5 Table — (PDF) [file pone.0286677.s005.pdf]

**S5 Table. Data for Fig 4.**

| Suc. conc. (%(w/v)) | $OD_{660\ nm}$ (1) | $OD_{660\ nm}$ (2) | $OD_{660\ nm}$ (3) | $OD_{660\ nm}$ (Ave.) | SD |
|---|---|---|---|---|---|
| 5 | 1.1482 | 1.1452 | 1.081 | 1.1248 | 0.03796156 |
| 10 | 1.274 | 1.3179 | 1.2167 | 1.269533333 | 0.050747644 |
| 20 | 1.2691 | 1.2719 | 1.2183 | 1.2531 | 0.030170184 |
| 30 | 1.169 | 1.214 | 1.1585 | 1.1805 | 0.029483046 |
| 40 | 0.8917 | 1.0224 | 0.8192 | 0.9111 | 0.102979755 |
| 50 | 0.4898 | 0.6042 | 0.6229 | 0.5723 | 0.072056297 |
| 60 | 0.0779 | 0.2287 | 0.1544 | 0.153666667 | 0.075402675 |
