# Supplementary material for: Philodulcilactobacillus myokoensis gen. nov., sp. nov., a fructophilic, acidophilic, and agar-phobic lactic acid bacterium isolated from fermented vegetable extracts
Source: PLoS One. 2023 Jun 21;18(6):e0286677. doi: 10.1371/journal.pone.0286677 (PMC10284405; doi:10.1371/journal.pone.0286677)
Supplement: S6 Table — (PDF) [file pone.0286677.s006.pdf]

S6 Table. Data for Fig 5.

Substrate conc. (%(w/v))	$OD_{660\ nm}$ (1)	$OD_{660\ nm}$ (2)	$OD_{660\ nm}$ (3)	$OD_{660\ nm}$ (Ave.)	SD
D-Fru. 20	0.2021	0.2085	0.198	0.202866667	0.005291818
D-Fru. 15＋D-Glu. 5	0.8023	0.8172	0.8317	0.817066667	0.014700454
D-Fru. 10＋D-Glu. 10	0.9443	0.9852	0.995	0.974833333	0.026892812
D-Fru. 5＋D-Glu. 15	1.1107	1.1522	1.0876	1.116833333	0.032733826
D-Glu. 20	0.201	0.2061	0.2068	0.204633333	0.003165965
Suc. 20	1.0209	1.0481	1.1145	1.061166667	0.048148659
