# Supplementary material for: Philodulcilactobacillus myokoensis gen. nov., sp. nov., a fructophilic, acidophilic, and agar-phobic lactic acid bacterium isolated from fermented vegetable extracts
Source: PLoS One. 2023 Jun 21;18(6):e0286677. doi: 10.1371/journal.pone.0286677 (PMC10284405; doi:10.1371/journal.pone.0286677)
Supplement: S7 Table — (PDF) [file pone.0286677.s007.pdf]

S7 Table. The pairwise amino acid identity of conserved genes (cAAI).

Genome A	Genes in A	Genome B	Genes in B	Orthologous genes[a]	cAAI	Orthologous fraction (%)[b]
GCF_001434095.1_protein	1313	GCF_001435995.1_protein	1427	816	61.97	62.15
GCF_003627035.1_protein	1151	GCF_001434095.1_protein	1313	791	62.18	68.72
GCF_003627035.1_protein	1151	GCF_001435995.1_protein	1427	923	69.03	80.19
GCF_004359375.1_protein	1498	GCF_003627035.1_protein	1151	750	60.46	65.16
GCF_004359375.1_protein	1498	GCF_001434095.1_protein	1313	720	58.46	54.84
GCF_004359375.1_protein	1498	GCF_001435995.1_protein	1427	775	60.37	54.31
GCF_001434175.1_protein	2932	GCF_004359375.1_protein	1498	736	56.41	49.13
GCF_001434175.1_protein	2932	GCF_003627035.1_protein	1151	838	59.57	72.81
GCF_001434175.1_protein	2932	GCF_001434095.1_protein	1313	882	58.36	67.17
GCF_001434175.1_protein	2932	GCF_001435995.1_protein	1427	908	59.3	63.63
GCF_001438685.1_protein	1395	GCF_001434175.1_protein	2932	889	57.63	63.73
GCF_001438685.1_protein	1395	GCF_004359375.1_protein	1498	698	57.49	50.04
GCF_001438685.1_protein	1395	GCF_003627035.1_protein	1151	777	61.2	67.51
GCF_001438685.1_protein	1395	GCF_001434095.1_protein	1313	978	68.58	74.49
GCF_001438685.1_protein	1395	GCF_001435995.1_protein	1427	817	61.02	58.57
GCF_002993965.1_protein	1538	GCF_001438685.1_protein	1395	824	60.57	59.07
GCF_002993965.1_protein	1538	GCF_001434175.1_protein	2932	900	58.73	58.52
GCF_002993965.1_protein	1538	GCF_004359375.1_protein	1498	795	59.73	53.07
GCF_002993965.1_protein	1538	GCF_003627035.1_protein	1151	986	74.34	85.66
GCF_002993965.1_protein	1538	GCF_001434095.1_protein	1313	827	61.64	62.99
GCF_002993965.1_protein	1538	GCF_001435995.1_protein	1427	1064	68.22	74.56
GCA_027923585.1_protein	1611	GCF_002993965.1_protein	1538	978	63.48	63.59
GCA_027923585.1_protein	1611	GCF_001438685.1_protein	1395	827	59.43	59.28
GCA_027923585.1_protein	1611	GCF_001434175.1_protein	2932	908	57.8	56.36
GCA_027923585.1_protein	1611	GCF_004359375.1_protein	1498	856	62.03	57.14
GCA_027923585.1_protein	1611	GCF_003627035.1_protein	1151	868	63.79	75.41

GCA_027923585.1_protein	1611	GCF_001434095.1_protein	1313	831	61.04	63.29
GCA_027923585.1_protein	1611	GCF_001435995.1_protein	1427	967	63.12	67.76
GCF_001281175.1_protein	1313	GCA_027923585.1_protein	1611	912	62.51	69.46
GCF_001281175.1_protein	1313	GCF_002993965.1_protein	1538	1027	72.9	78.22
GCF_001281175.1_protein	1313	GCF_001438685.1_protein	1395	818	60.23	62.30
GCF_001281175.1_protein	1313	GCF_001434175.1_protein	2932	881	58.82	67.10
GCF_001281175.1_protein	1313	GCF_004359375.1_protein	1498	750	59.61	57.12
GCF_001281175.1_protein	1313	GCF_003627035.1_protein	1151	1031	78.58	89.57
GCF_001281175.1_protein	1313	GCF_001434095.1_protein	1313	811	61.31	61.77
GCF_001281175.1_protein	1313	GCF_001435995.1_protein	1427	981	67.73	74.71
GCF_001436645.1_protein	1308	GCF_023380205.1_protein	1635	803	59.02	61.39
GCF_001436645.1_protein	1308	GCF_001281175.1_protein	1313	801	58.29	61.24
GCF_001436645.1_protein	1308	GCA_027923585.1_protein	1611	779	58	59.56
GCF_001436645.1_protein	1308	GCF_002993965.1_protein	1538	793	58.31	60.63
GCF_001436645.1_protein	1308	GCF_001438685.1_protein	1395	990	69.42	75.69
GCF_001436645.1_protein	1308	GCF_001434175.1_protein	2932	830	57.07	63.46
GCF_001436645.1_protein	1308	GCF_004359375.1_protein	1498	679	56.2	51.91
GCF_001436645.1_protein	1308	GCF_003627035.1_protein	1151	752	59.49	65.33
GCF_001436645.1_protein	1308	GCF_001434095.1_protein	1313	941	64.09	71.94
GCF_001436645.1_protein	1308	GCF_001435995.1_protein	1427	791	58.78	60.47
GCF_023380205.1_protein	1635	GCF_001281175.1_protein	1313	976	64.04	74.33
GCF_023380205.1_protein	1635	GCA_027923585.1_protein	1611	1031	62.87	64.00
GCF_023380205.1_protein	1635	GCF_002993965.1_protein	1538	1002	64.14	65.15
GCF_023380205.1_protein	1635	GCF_001438685.1_protein	1395	851	59.92	61.00
GCF_023380205.1_protein	1635	GCF_001434175.1_protein	2932	979	59.3	59.88
GCF_023380205.1_protein	1635	GCF_004359375.1_protein	1498	833	60.5	55.61
GCF_023380205.1_protein	1635	GCF_003627035.1_protein	1151	899	64.78	78.11
GCF_023380205.1_protein	1635	GCF_001434095.1_protein	1313	849	61.36	64.66
GCF_023380205.1_protein	1635	GCF_001435995.1_protein	1427	962	64.45	67.41

GCF_001436035.1_protein	1212	GCF_002993975.1_protein	1403	762	60.44	62.87
GCF_001436035.1_protein	1212	GCF_001436645.1_protein	1308	907	68.04	74.83
GCF_001436035.1_protein	1212	GCF_023380205.1_protein	1635	801	60.16	66.09
GCF_001436035.1_protein	1212	GCF_001281175.1_protein	1313	781	60.29	64.44
GCF_001436035.1_protein	1212	GCA_027923585.1_protein	1611	782	59.51	64.52
GCF_001436035.1_protein	1212	GCF_002993965.1_protein	1538	788	60.26	65.02
GCF_001436035.1_protein	1212	GCF_001438685.1_protein	1395	959	75.09	79.13
GCF_001436035.1_protein	1212	GCF_001434175.1_protein	2932	818	57.86	67.49
GCF_001436035.1_protein	1212	GCF_004359375.1_protein	1498	686	57.16	56.60
GCF_001436035.1_protein	1212	GCF_003627035.1_protein	1151	754	61.06	65.51
GCF_001436035.1_protein	1212	GCF_001434095.1_protein	1313	891	67.39	73.51
GCF_001436035.1_protein	1212	GCF_001435995.1_protein	1427	796	60.3	65.68
GCF_002993975.1_protein	1403	GCF_001436645.1_protein	1308	758	58.74	57.95
GCF_002993975.1_protein	1403	GCF_023380205.1_protein	1635	962	64.66	68.57
GCF_002993975.1_protein	1403	GCF_001281175.1_protein	1313	999	73.06	76.09
GCF_002993975.1_protein	1403	GCA_027923585.1_protein	1611	940	63.67	67.00
GCF_002993975.1_protein	1403	GCF_002993965.1_protein	1538	1215	94.18	86.60
GCF_002993975.1_protein	1403	GCF_001438685.1_protein	1395	800	60.73	57.35
GCF_002993975.1_protein	1403	GCF_001434175.1_protein	2932	874	59.27	62.30
GCF_002993975.1_protein	1403	GCF_004359375.1_protein	1498	763	60.06	54.38
GCF_002993975.1_protein	1403	GCF_003627035.1_protein	1151	953	74.54	82.80
GCF_002993975.1_protein	1403	GCF_001434095.1_protein	1313	804	61.8	61.23
GCF_002993975.1_protein	1403	GCF_001435995.1_protein	1427	1004	68.83	71.56
GCF_001433825.1_protein	1340	GCF_001436035.1_protein	1212	803	60.23	66.25
GCF_001433825.1_protein	1340	GCF_002993975.1_protein	1403	1027	73.58	76.64
GCF_001433825.1_protein	1340	GCF_001436645.1_protein	1308	798	58.71	61.01
GCF_001433825.1_protein	1340	GCF_023380205.1_protein	1635	1004	64.35	74.93
GCF_001433825.1_protein	1340	GCF_001281175.1_protein	1313	1141	83.04	86.90
GCF_001433825.1_protein	1340	GCA_027923585.1_protein	1611	957	62.57	71.42

GCF_001433825.1_protein	1340	GCF_002993965.1_protein	1538	1067	73.11	79.63
GCF_001433825.1_protein	1340	GCF_001438685.1_protein	1395	828	60.67	61.79
GCF_001433825.1_protein	1340	GCF_001434175.1_protein	2932	912	58.94	68.06
GCF_001433825.1_protein	1340	GCF_004359375.1_protein	1498	783	59.47	58.43
GCF_001433825.1_protein	1340	GCF_003627035.1_protein	1151	1045	80.22	90.79
GCF_001433825.1_protein	1340	GCF_001434095.1_protein	1313	832	61.53	63.37
GCF_001433825.1_protein	1340	GCF_001435995.1_protein	1427	995	68.08	74.25
GCF_002994005.1_protein	1507	GCF_001433825.1_protein	1340	1075	73.13	80.22
GCF_002994005.1_protein	1507	GCF_001436035.1_protein	1212	791	60.34	65.26
GCF_002994005.1_protein	1507	GCF_002993975.1_protein	1403	1204	90.15	85.82
GCF_002994005.1_protein	1507	GCF_001436645.1_protein	1308	798	58.39	61.01
GCF_002994005.1_protein	1507	GCF_023380205.1_protein	1635	1013	64.15	67.22
GCF_002994005.1_protein	1507	GCF_001281175.1_protein	1313	1044	72.62	79.51
GCF_002994005.1_protein	1507	GCA_027923585.1_protein	1611	988	63.48	65.56
GCF_002994005.1_protein	1507	GCF_002993965.1_protein	1538	1244	90.46	82.55
GCF_002994005.1_protein	1507	GCF_001438685.1_protein	1395	823	60.65	59.00
GCF_002994005.1_protein	1507	GCF_001434175.1_protein	2932	900	58.64	59.72
GCF_002994005.1_protein	1507	GCF_004359375.1_protein	1498	787	60.12	52.54
GCF_002994005.1_protein	1507	GCF_003627035.1_protein	1151	998	74.16	86.71
GCF_002994005.1_protein	1507	GCF_001434095.1_protein	1313	833	61.63	63.44
GCF_002994005.1_protein	1507	GCF_001435995.1_protein	1427	1034	68.62	72.46
GCF_023380225.1_protein	1468	GCF_002994005.1_protein	1507	1191	82.1	81.13
GCF_023380225.1_protein	1468	GCF_001433825.1_protein	1340	1056	73.49	78.81
GCF_023380225.1_protein	1468	GCF_001436035.1_protein	1212	795	60.29	65.59
GCF_023380225.1_protein	1468	GCF_002993975.1_protein	1403	1151	82.68	82.04
GCF_023380225.1_protein	1468	GCF_001436645.1_protein	1308	798	58.43	61.01
GCF_023380225.1_protein	1468	GCF_023380205.1_protein	1635	998	64.39	67.98
GCF_023380225.1_protein	1468	GCF_001281175.1_protein	1313	1052	72.78	80.12
GCF_023380225.1_protein	1468	GCA_027923585.1_protein	1611	958	63.44	65.26

GCF_023380225.1_protein	1468	GCF_002993965.1_protein	1538	1213	81.78	82.63
GCF_023380225.1_protein	1468	GCF_001438685.1_protein	1395	824	60.57	59.07
GCF_023380225.1_protein	1468	GCF_001434175.1_protein	2932	893	58.67	60.83
GCF_023380225.1_protein	1468	GCF_004359375.1_protein	1498	779	60.06	53.07
GCF_023380225.1_protein	1468	GCF_003627035.1_protein	1151	986	74.1	85.66
GCF_023380225.1_protein	1468	GCF_001434095.1_protein	1313	828	61.6	63.06
GCF_023380225.1_protein	1468	GCF_001435995.1_protein	1427	1051	68.24	73.65
GCF_016861895.1_protein	1405	GCF_023380225.1_protein	1468	1071	73.09	76.23
GCF_016861895.1_protein	1405	GCF_002994005.1_protein	1507	1081	73.04	76.94
GCF_016861895.1_protein	1405	GCF_001433825.1_protein	1340	1242	94.86	92.69
GCF_016861895.1_protein	1405	GCF_001436035.1_protein	1212	803	60.37	66.25
GCF_016861895.1_protein	1405	GCF_002993975.1_protein	1403	1028	73.5	73.27
GCF_016861895.1_protein	1405	GCF_001436645.1_protein	1308	819	58.37	62.61
GCF_016861895.1_protein	1405	GCF_023380205.1_protein	1635	1030	64.42	73.31
GCF_016861895.1_protein	1405	GCF_001281175.1_protein	1313	1155	82.75	87.97
GCF_016861895.1_protein	1405	GCA_027923585.1_protein	1611	970	62.57	69.04
GCF_016861895.1_protein	1405	GCF_002993965.1_protein	1538	1072	73.12	76.30
GCF_016861895.1_protein	1405	GCF_001438685.1_protein	1395	848	60.4	60.79
GCF_016861895.1_protein	1405	GCF_001434175.1_protein	2932	934	58.84	66.48
GCF_016861895.1_protein	1405	GCF_004359375.1_protein	1498	793	59.35	56.44
GCF_016861895.1_protein	1405	GCF_003627035.1_protein	1151	1055	80.03	91.66
GCF_016861895.1_protein	1405	GCF_001434095.1_protein	1313	841	61.42	64.05
GCF_016861895.1_protein	1405	GCF_001435995.1_protein	1427	1005	67.79	71.53

cAAI, amino acid identity of conserved genes

[a]: Orthologous genes identified by CompareM.

[b]: The orthologous fraction between the two genomes is defined as the number of orthologous genes divided by the minimum number of genes in either genome.
